# Supplementary material for: Rapid turnover of life-cycle-related genes in the brown algae
Source: Genome Biol. 2019 Feb 14;20:35. doi: 10.1186/s13059-019-1630-6 (PMC6374913; doi:10.1186/s13059-019-1630-6)
Supplement: Supplementary file 3 — Protein alignments, corresponding Gblocks html file and CDS sequences for the lineage conserved gametophyte-biased genes. (ZIP 2109 kb) [file 13059_2019_1630_MOESM3_ESM.zip › Con_GA_alignments/Ec-01_003790.aa_ali.fasta-gb.htm]

Ec-01\_003790.aa.fasta.trans.aa\_ali.fasta


## Gblocks 0.91b Results

Processed file: **Ec-01\_003790.aa.fasta.trans.aa\_ali.fasta**  
Number of sequences: **2**  
Alignment assumed to be: **Protein**  
New number of positions: **676** (selected positions are underlined in blue)

```
                         10        20        30        40        50        60
                 =========+=========+=========+=========+=========+=========+
Ec-01_003790     MGVPMFGNPESPVGFGLGGDAAQLSNGGGEDGINGDGRGVRETLADTIYPNSSKAPQNND
MSTRG.54064.1    ------------------------------------------------------------
                                                                             


                         70        80        90       100       110       120
                 =========+=========+=========+=========+=========+=========+
Ec-01_003790     DGNDHAVGMGSRRGFGEGGQGASDDAGSRSRRMFDGSRVAEPDNLQIVEAGEGADIIPGV
MSTRG.54064.1    ------------------------------------------------------------
                                                                             


                        130       140       150       160       170       180
                 =========+=========+=========+=========+=========+=========+
Ec-01_003790     DDLPVFANDQSKALNDEIKVREKKVAQATNALADNRERVRIMEEHLKNVRQEVGHTNALM
MSTRG.54064.1    ----------------------------------------------MCFRLTCCRTPATS
                                                                  ###########


                        190       200       210       220       230       240
                 =========+=========+=========+=========+=========+=========+
Ec-01_003790     AAKKK--EIATEEHLCALAAREAGRYTHDARRFVVEAEEAQDKLNGVQNAIFGANEEMDR
MSTRG.54064.1    AETKRWPCVPSRNILCTFDLSRSRRSKHES------------------------------
                 ####          ##############                                


                        250       260       270       280       290       300
                 =========+=========+=========+=========+=========+=========+
Ec-01_003790     FKLQMNWNQEELEQWALAAKQKEEDNLALQKYARADDVKIKDLSLQVERLSKSVVARRAE
MSTRG.54064.1    ----------------------------------------------------TT------
                                                                             


                        310       320       330       340       350       360
                 =========+=========+=========+=========+=========+=========+
Ec-01_003790     LQNLEAESSAKQVELDNTAADFRRLHAERQDLVRQWQNAIDGMRRRDEEINAVGEKYAAA
MSTRG.54064.1    ---HRRSCKLAQVELDNTATDFRQLHAERQDLVRQWQNAIDGMRRRDEEINAVGEKYAAA
                            #################################################


                        370       380       390       400       410       420
                 =========+=========+=========+=========+=========+=========+
Ec-01_003790     RQVRATRIETLAQNAARLKMQRNDNKEVQGSIENLERTVQRQREEAQAAQKRLEEFRDVL
MSTRG.54064.1    RQVRASRIETLAQNAARLKMQKNDNEEVQASIENLERSVQRQREESQAAQKRLEEFRDVL
                 ############################################################


                        430       440       450       460       470       480
                 =========+=========+=========+=========+=========+=========+
Ec-01_003790     EVVKNTLGNGAGALARKRGENANLTAQIEEKRVGLDTARRKYQISKQRVETETGNTNKVE
MSTRG.54064.1    EVVKNTLGNGAGALSRKRGENANLNAQIEEKRVGLETARRKYQMSKQRVETETGNTNKVE
                 ############################################################


                        490       500       510       520       530       540
                 =========+=========+=========+=========+=========+=========+
Ec-01_003790     ATAKSAEEELQQREAELKASKKRLATAKELMFRDSQKLYALRQEEANFIAEICGAQATMK
MSTRG.54064.1    ATAKSAEEDLQKREAELRASKKRLATVKELMFRDSQKLYASRQEEANLIAEICGAQATMK
                 ############################################################


                        550       560       570       580       590       600
                 =========+=========+=========+=========+=========+=========+
Ec-01_003790     NLQTKVHALDQEQTRQQELIYSAEFQIQQMERKVARGMGERTDEEKSELNAKIRELEGEV
MSTRG.54064.1    NLQTKVHALDQEQTRQQELIYSAEFQIQQMERKVARGMGERTDEEKSQLNTKIRELEGEV
                 ############################################################


                        610       620       630       640       650       660
                 =========+=========+=========+=========+=========+=========+
Ec-01_003790     DVMREEKKMLTGQARKLNNELRASLRRREAASAQQAELNEAINELELECRAAEGDVKRES
MSTRG.54064.1    EILREEKKMLTGQARKLNNELRASLRRKEVASAQRAELDEAINELELECRAAEGDVRRES
                 ############################################################


                        670       680       690       700       710       720
                 =========+=========+=========+=========+=========+=========+
Ec-01_003790     QMKEELMVSNDVMRLEVKRLRDTLNGAADEVFSLENRRQQLAMSMKERKAEIQVHWDLQR
MSTRG.54064.1    QTKEELMVSNDVMRLEVKRLRDTLNGAADEVFSLENRRQQLAMSMKERKAEIQVHWDLQR
                 ############################################################


                        730       740       750       760       770       780
                 =========+=========+=========+=========+=========+=========+
Ec-01_003790     ATLRAAEEERHRVQLELGDRRQAVEKLRAKYETLAKGFAGAGAGELGGDHAGGGSSQAYF
MSTRG.54064.1    ATLRAAEEERHRVQLELGDRRQAVEKLRAKYETLAKGFAGAGGGEEGGDRTGAGSSQAYF
                 ############################################################


                        790       800       810       820       830       840
                 =========+=========+=========+=========+=========+=========+
Ec-01_003790     IIAAAQKREELQREGDELDQEIR-------------------------SFQRADPGCKDA
MSTRG.54064.1    IIAAAQKREELQREGDELDQEIRRCEREIRALESTLRHLNARNVDFRVSFQRADPGSKDA
                 #######################                         ############


                        850       860       870       880       890       900
                 =========+=========+=========+=========+=========+=========+
Ec-01_003790     DALRSLEARAKAAQDGLFRRKKELQRAQTDHEEDLARLDGAKARAARLDEQNAQLDSVRV
MSTRG.54064.1    DALRSLEARAKAAQDGLFRRKKELQRAQTDHEEDLARLDGAKARAARLDEQNAQLESVQA
                 ############################################################


                        910       920       930       940       950       960
                 =========+=========+=========+=========+=========+=========+
Ec-01_003790     QAKAQLDAQRQALTRAGERLASERAMHRRAPGVGGGGRSDRDGGVETMVPTADDVTPAPV
MSTRG.54064.1    QAEAQLKAQRDSLRRAEERLASERTMHRKAPGAGAG--SGGGVGAETVVSTADGVTPAPV
                 ####################################  ######################


                        970       980       990      1000      1010      1020
                 =========+=========+=========+=========+=========+=========+
Ec-01_003790     AGGTATAEARDNGGGLIAALVGRGGETLTEKALRASTLGDTASGVLYTLGQLAREFPEMH
MSTRG.54064.1    AGGTAMAGSNNNGGPAVAALVGRGG-----------------------------------
                 #########################                                   


                       1030      1040      1050      1060      1070      1080
                 =========+=========+=========+=========+=========+=========+
Ec-01_003790     DALKISLHLKKLKVPPAPPAHGPASGVAAGASKTGRASSAEGSRAGSESSSHVGGASVRS
MSTRG.54064.1    ------------------------------------------------------------
                                                                             


                       1090      1100      1110      1120
                 =========+=========+=========+=========+=
Ec-01_003790     STSGGGMRGGSLGDRRGSRAGGGGSVAGEGISPMRLFEAPI
MSTRG.54064.1    -----------------------------------------
```

```
Parameters used
Minimum Number Of Sequences For A Conserved Position: 2
Minimum Number Of Sequences For A Flanking Position: 2
Maximum Number Of Contiguous Nonconserved Positions: 8
Minimum Length Of A Block: 10
Allowed Gap Positions: None
Use Similarity Matrices: Yes
```

```
Flank positions of the 5 selected block(s)
Flanks: [170  184]  [195  208]  [312  803]  [829  936]  [939  985]  

New number of positions in Ec-01_003790.aa.fasta.trans.aa_ali.fasta-gb:  676  (60% of the original 1121 positions)
```
